# Geographical Accessibility of the Sarcoma Referral Networks in France. Intermediate Results from the IGéAS Research Program

**DOI:** 10.3390/ijerph15102204

**Published:** 2018-10-10

**Authors:** Yohan Fayet, Jean-Michel Coindre, Cécile Dalban, François Gouin, Gonzague De Pinieux, Fadila Farsi, Françoise Ducimetière, Claire Chemin-Airiau, Myriam Jean-Denis, Sylvie Chabaud, Jean-Yves Blay, Isabelle Ray-Coquard

**Affiliations:** 1 Equipe Evaluation Médicale et Sarcomes, Centre Léon Bérard, Université Claude Bernard Lyon 1, HESPER EA 7425, 69008 Lyon, France; 2 Institut Bergonié, RRePS Network, 33000 Bordeaux, France; j.coindre@bordeaux.unicancer.fr; 3 Direction de la Recherche Clinique et de l’Innovation, Centre Léon Bérard, 69008 Lyon, France; cecile.dalban@lyon.unicancer.fr (C.D.); sylvie.chabaud@lyon.unicancer.fr (S.C.); 4 Centre Hospitalier Universitaire Nantes, ResOs Network, 44000 Nantes, France; francois.gouin@chu-nantes.fr; 5 Centre Hospitalier Universitaire Tours, ResOs Network, 37000 Tours, France; gonzague.dubouexic@univ-tours.fr; 6 Réseau Régional de Cancérologie Auvergne–Rhône–Alpes, 69008 Lyon, France; Fadila.FARSI@espacecancer.sante-ra.fr; 7 Equipe Evaluation Médicale et Sarcomes, Centre Léon Bérard, Netsarc RRePS ResOs Networks, 69008 Lyon, France; francoise.ducimetiere@lyon.unicancer.fr (F.D.); claire.chemin@lyon.unicancer.fr (C.C.-A.); myriam.jean-denis@lyon.unicancer.fr (M.J.-D.); 8 Centre Léon Bérard, Netsarc Network, Université Claude Bernard Lyon 1, 69008 Lyon, France; jean-yves.blay@lyon.unicancer.fr; 9 Centre Léon Bérard, Netsarc Network, Université Claude Bernard Lyon 1, HESPER EA 7425, 69008 Lyon; France; isabelle.ray-coquard@lyon.unicancer.fr

**Keywords:** sarcoma, referral networks, cancer inequalities, cancer care quality, multidisciplinary tumor board, histological review, health care access

## Abstract

Rare cancer patients face lower survival and experience delays in diagnosis and therapeutic mismanagement. Considering the specificities of rare cancers, referral networks have been implemented in France to improve the management and survival of patients. The IGéAS research program aims to assess the networks’ ability to reduce inequalities. Data analysis of the IGéAS cohort (*n* = 20,590, sarcoma diagnosed between 2011 and 2014) by gathering medical data and geographical index will identify risk factors associated with the belated access to expertise or with no access to expertise. Intermediate results show that referral networks give sarcoma patients access to sarcoma expertise despite the remoteness of some of them. Regional expert centers mostly receive requests from within their area while national referral centers receive requests from the whole country. Delays in the access to expertise may be reduced by making outside practitioners more sensitive to the issues of rare cancers. The perception and involvement of outside practitioners in this device will be assessed using a qualitative survey. All the results are discussed and will contribute to design guidelines to improve early access to expertise and reduce inequalities. Results of the IGéAS research program may contribute to the assessment of referral sarcoma networks and provide some useful lessons to improve cancer care management.

## 1. Introduction

The management of rare cancers has a higher proportion of deaths than common cancers and poses specific problems, including delays in diagnosis due to less diagnostic precision and therapeutic mismanagement. Patients with rare cancers and their primary physician have difficulties in accessing information on diagnosis and treatment [1]. The experience of rare cancers by the medical team is critical for patient outcome. Sarcomas, which represent 1% of all cancers, are typically representative of features of these rare cancers. Indeed, recent studies on exhaustive population-based series estimate that only 40% to 50% of patients with sarcoma are treated according to the clinical guidelines for localized disease; the remaining patients receive treatment or management that is not performed according to clinical practice guidelines (CPG) and have a worse survival as a result [2,3]. Several studies have also investigated the frequency of diagnostic inaccuracies. When a central review was done systematically, 30% of the histology diagnoses were substantially modified [4]. Overall, approximatively 10% of patients will have a complete modification of management from the initial to the rectified diagnosis. Finally, the rarity of these cancers induces a delayed diagnosis and/or treatment and contributes to the strong feelings of isolation experienced by patients and those close to them. Provision of information to patients and their relatives must be facilitated. The improvement of management to increase the survival of rare cancers requires patient referrals towards expert centers, specializing in these cancers [5,6,7,8].

Three national referral sarcoma networks have been supported since 2010 by the French National Cancer Institute (INCa) and were certified in 2014:

- RRePS: Pathological referral network for soft tissue and visceral sarcomas
- ResOs: Pathological and clinical referral network for bone sarcomas
- Netsarc: Clinical referral network for soft tissue and visceral sarcomas

Centers belonging to pathological networks ensure a histological review of any new cases of soft tissue, viscera, and bone sarcomas to confirm the initial diagnosis and ensure optimal management. Centers belonging to clinical networks host a multidisciplinary tumor board (MTB) on sarcoma, which guides practitioners and provides advice for the management of their patients. The networks comprise national referral centers and regional expert centers. National referral centers are responsible for structuring the national network, arranging the double reading of tumor specimens, structuring referral multidisciplinary consultative meetings, contributing to clinical research on these rare cancers as well as drafting recommendations for good practice and their dissemination throughout the network. These centers also have to establish a national database to contribute to the observation of these cancers, organize training for all health professionals involved, work in collaboration with the patient associations, and provide relevant information to patients and their relatives. The networks’ data show that the frequency of discordances between local and central diagnosis of sarcoma versus non-sarcoma was around 25% for a review request and 10% for systematic review [9]. In addition to the positive impact of centralized histological reviews on the quality of diagnosis, medico-economic studies have demonstrated that histological reviews lower the cost of disease management for the French Social Security [10]. National sarcoma network structuring seems to improve practices for this implementation and data analysis has revealed a better quality of initial management (higher use of imaging, biopsy before resection) within the Netsarc network [7,11]. The French experience of a centralized organization of sarcoma management has already been set up in several foreign countries, particularly Scandinavian countries and the UK [12,13]. In the UK, expert centers already play a key role in the diagnosis and complex multimodality treatments of sarcoma.

The rare cancers referral organization gives the opportunity to all sarcoma patients to benefit from pathological and clinical expertise without forcing them to travel to the largest cities in France where this expertise is available. Indeed, expert centers analyze the patient’s specimen and medical file before requiring, if necessary, the patient referral to an expert center. This may reduce geographical inequalities during cancer management, bringing local practitioners’ knowledge about the pathology, and the most suitable therapeutic indications. Standardized mortality rates vary two fold in different regions of France [14]. Research on geographical inequalities in cancer has particularly sought to assess the impact of health care services and their accessibility. Indeed, the quality of cancer care and survival correlates to the number of patients managed and/or level of specialization of units [15,16]. These structures are often located in larger cities and are not accessible or visible for all patients, even more so for remote patients. Urban–rural inequalities have been well studied for survival, but not so much for cancer management [17]. Several French and international studies have shown a lower access to expertise for people living in socio-economically deprived areas, in rural communities, and in places remote from specialized centers [18,19].

Thus, the literature reports that access to a referral care center could be strongly determined by distance or area deprivation. The clinical consequences of this difficult access to expertise for several populations could be particularly serious in the case of sarcoma, considering the importance of first treatment on survival. This has been suggested by the geographical analysis of a sarcoma cohort (*n* = 698, diagnosis 2005–2006) in the Rhône–Alpes region which compared patient management and survival according to their geographical context before the implementation of any national network. Results report the loss of survival chances during cancer management for patients living in rural and urban deprived areas [20]. Following the results in the Rhône–Alpes region, which represented 10% of the French population, it was hypothesized that geographical inequalities between sarcoma patients in France may reflect a differential access to expertise. The Geographical inequalities in the access to cancer expertise. An evaluation of the French Sarcoma networks (IGéAS) research program aims to assess the ability of the national sarcoma networks to reduce inequalities in access to expertise. The analysis of the activity of the national sarcoma networks and the geographical coverage of expert centers is needed to assess the benefit of this innovative organization to remote patients and its ability to reduce geographical inequalities.

## 2. Materials and Methods

### 2.1. National Network Databases

A website collecting all patients with a pathological review and all patients discussed in the sarcoma specialized MTB in referral network centers was generated in 2010). The databases contain 60 items divided into four themes: characteristics of the patient and tumor, diagnosis and review, key steps in management and follow-up, and successive presentations of the file and decision making at MTB. The patient’s place of residence (French “Commune”, municipal scale) at diagnosis is recorded. The prospective collection of the address of the patient, diagnosis and clinical data, place of surgery and patient follow-up are prospectively implemented in the databases. The yearly follow-up of these national databases helps monitor the evolution of clinical practices. A quality assurance program has been established for these databases to ensure the quality of medical data recorded.

### 2.2. IGéAS Cohort Extraction

The inclusion criteria of the IGéAS cohort are:

- Patient living in France at time of diagnosis;
- Diagnoses of sarcoma/GIST/desmoid tumor/intermediate malignity tumor between the 1 January 2011 and the 31 December 2014;
- Patient who benefits from a pathological review or a sarcoma MTB discussion in an expert center belonging to the sarcoma referral networks.

The following data were extracted from the database: patient (sex, age, past medical history, address at diagnosis) and tumor (size, grade, stage, localization, type) characteristics and management (clinical situation at RCP). Considering the inclusion criteria, the IGéAS cohort was comprised of 20,590 patients amongst which 488 patients came from overseas territories (French DOM–TOM). Having access to expertise before surgery or after relapse as not considered to be the same, especially for sarcomas, which are mainly aggressive tumors. It was then important to focus our analysis on the ability of the networks to provide early access to expertise to improve patient prognosis. In accordance with sarcoma guidelines and the opinion of sarcoma experts, we considered optimal access to pathological expertise if a review was performed within 30 days of sampling, and optimal access to clinical expertise if the first sarcoma MTB was performed before the first surgery.

### 2.3. Flow Maps

Mapping of the flows between patient location and place of the histological review or sarcoma MTB discussion was performed thanks to QGis software (QGis, Grüt, Swiss). Each line represents a relation between the commune and expert center.

Numerous communes (mostly urban communes) have several sarcoma patients in the IGéAS cohort and patients of the same commune may be addressed to different expert centers. The size of the flow was calculated according the number of patients comprising each relation. To improve the map reading, we separated soft-tissue, bone, and visceral sarcoma flows as these sarcomas are specific and require different expertise. If necessary, we also distinguished between flows to national referral centers, which present a higher activity, and flows to regional expert centers.

During our study period, there were 21 clinical expert centers and 38 pathological expert centers. Some of these pathological expert centers were in the same urban areas and even belonged to the same hospital organization (for example, in Paris). To improve the map reading, we decided to gather all flows coming to the same city even if they went to different expert centers because our aim was to describe the geographical organization of these networks instead of focusing on the specific activity of some expert centers. The size of the flows was proportional to the number of patients from a commune who were referred to a city with sarcoma expertise. Flows were also colored according to the city requested.

### 2.4. Measuring the Accessibility of the Networks

To measure the patient’s remoteness to expertise, the calculation of the travel time to the closest expert center was performed using Odomatrix^®^ software (Source: ODOMATRIX, INRA UMR1041 CESAER, from IGN Route500^®^ IGN, Saint-Mandé, France). The travel time was calculated from the town hall of each commune as the complete address of the patients was impossible to collect. Nonetheless, even if the calculation from the town hall address was less accurate than that from the patient’s home address, the difference (few minutes at best) should not strongly influence the results as it was for a primary care access study. Median and average travel times to the closest expert center were calculated to assess the overall distance of the French population from the expert centers. Average travel time (in minutes) by quintiles was intended to measure the travel time gaps across the French population. Maps of this travel time to the closest expert center were performed using QGis software to describe geographical inequalities and identify remote areas, which may benefit from the networks’ scheme.

## 3. Results

### 3.1. IGéAS Cohort Description

Considering the inclusion criteria, the IGéAS cohort was comprised of 20,590 patients and included about as many men as women (Table 1). A total of 2.4% of the population came from overseas territories (French DOM–TOM). Most of the patients included in the cohort had been diagnosed for soft tissue sarcoma, which corresponded to the distribution by subtypes of sarcomas that are usually published in the literature. Access to referral networks is gradually increasing with more and more patients benefiting from a pathological review or MTB discussion each year in a referral or expert center. Almost a thousand more patients had access to expertise in 2014 (*n* = 5583) in comparison to 2011 (*n* = 4594). This significant increase most likely reflects an improvement of medical practices in sarcoma management and a better knowledge of the networks across the country. Fifty-eight percent of the patients had optimal access to pathological expertise (less than 30 days between date of sampling and date of review) and 31% of the patients benefited from optimal access to clinical expertise (sarcoma MTB before first surgery).

### 3.2. Geographical Coverage of the Sarcoma Referral Networks

**Figure 1:**
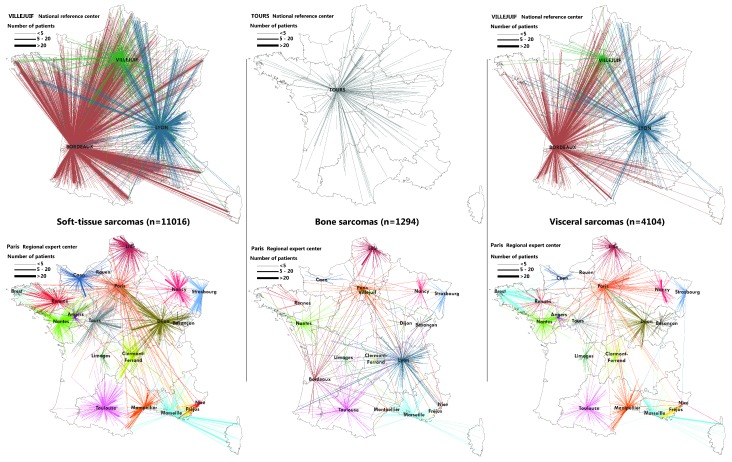
Sarcoma patients’ dwelling place in mainland France (diagnoses between 2011 and 2014) and place of their specimen’s review.

**Figure 2:**
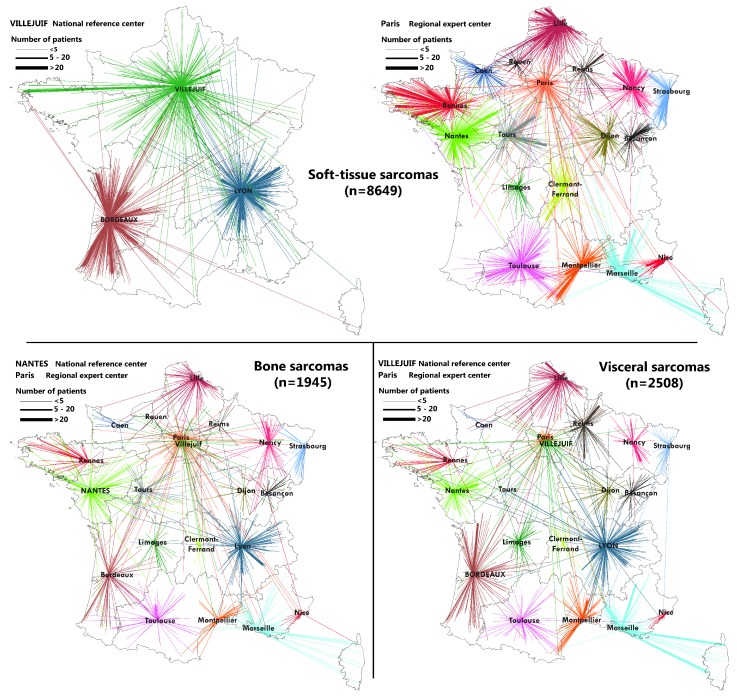
Sarcoma patients’ dwelling place in mainland France (diagnosed between 2011 and 2014) and place of their expert MTB discussion.

Figure 1 represents the flows between patient location and the histological review center and Figure 2 deals with access to the sarcoma expert Multidisciplinary Tumor Board. Maps illustrate the regional influence of expert centers, which are mostly called on by their regional colleagues. As expected, national referral centers, called upon by institutions/establishments from across the whole country, presented a larger area of influence. Even if sarcoma expertise was concentrated in a few dozen French cities, the maps point out that remote patients may benefit from this organization. This national coverage of the referral sarcoma networks has to be considered as a crucial asset of this organization.

Some local expertise also appeared out of the comparative analysis of these maps. It could be seen, for example, that numerous soft-tissue sarcoma patients living in the Paris area benefitted from a review in Dijon, while we did not observe this attractiveness for bone and visceral sarcomas. This suggests that practitioners may not systematically refer the tumor specimen or clinical file of their patient to the same expert center. In some cases, they chose not to call upon their regional expert center, but an expert center further away, considering the patient or tumor characteristics and the specialist expertise of this further center. This reports on the ability of some practitioners to identify specific expertise and adapt their request to their patient’s characteristics. This was also illustrated by the flows to national referral centers.

Special flows between some areas and centers (visceral sarcoma histological reviews in Paris of patients from eastern France, soft-tissue sarcoma MTB in Villejuif of Brest area patients) also reflect the practitioners’ preference pathways, which may be linked to medical collaboration between hospitals and personal relationships between practitioners.

### 3.3. Analysis of the Patients’ Remoteness to Sarcoma Expertise

Remoteness to expertise was estimated in terms of travel time to the closest expert center. Table 2 presents the median and average travel time to the closest pathological or clinical expert centers. We also describe the average travel time across quintiles.

Considering the high number of communes with pathological expert centers, the median and average travel time was lower for pathological expertise in comparison to clinical expertise, even if the differences were not significant. French communes are situated, on average, 80 min from the closest pathological expertise and 82 min from the closest clinical expertise. The quintile calculations show strong inequalities between French communes in terms of remoteness to sarcoma expertise. The average travel time to the closest pathological or clinical expert centers as more than three times higher for the furthest quintile in comparison to the closest.

**Figure 3:**
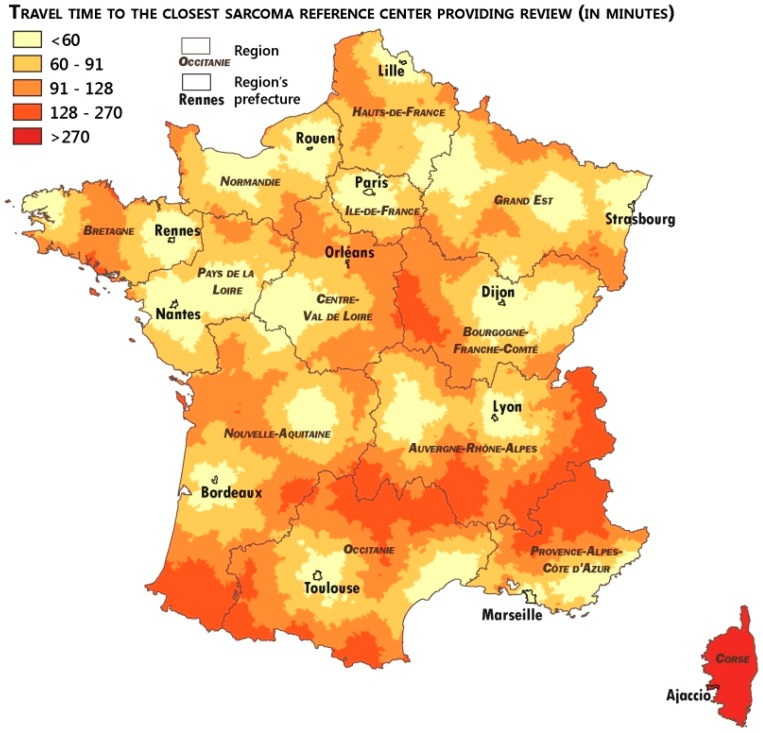
Access to pathological clinical expertise by mainland French communes.

**Figure 4:**
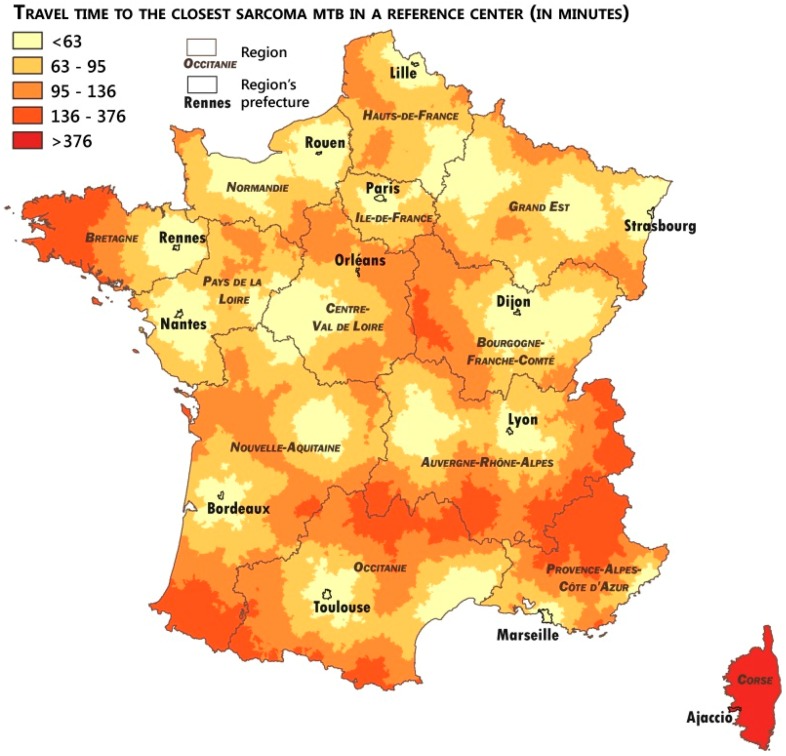
Access to sarcoma clinical expertise by mainland French communes.

Figure 3 and Figure 4 describe the geographical inequalities in the access to sarcoma pathological and clinical expertise. We observed a quite similar situation for these two factors. The Corse Region experienced a specific situation because the island does not have a pathological or clinical expert center. Most of the areas highly remote to sarcoma expertise (dark orange areas in the maps, with a travel time higher than 128 min to pathological expertise and 136 min to clinical expertise) were mountain areas (the Alps in the southeast of France, the Pyrénées in the southwest, the Massif–Central in the south–center). This situation of remoteness of the mountain areas was not specific to the sarcoma care access and reflects the impact of the mountain roads on travel time.

Remoteness to clinical expertise of areas in the west of the Bretagne Region, shown in Figure 4, could be nuanced by flows maps (Figure 2), which show the strong referral of sarcoma patients to the Rennes and Villejuif centers. It proves that even if some areas appear to be disadvantaged by the concentration and remoteness to expert centers, good practices by the local practitioners, who referred their patients to expert centers through referral sarcoma networks, could improve their patients’ access to expertise.

## 4. Discussion

The results provide new information regarding the activity and function of these networks. Data show the gradual improvement of the networks’ activity, which underlines their ability to influence medical practices in sarcoma management and benefit an ever-increasing number of sarcoma patients in France. This may be seen as a result of the warnings of the referral sarcoma networks and the French Sarcoma Group (GSF-GETO) to non-expert practitioners about sarcoma management. The data from the networks also enable the assessment of the earliness of the access to expertise and we observed better outcomes for the histological review than the MTB. While access to pathological expertise only depends on the pathologists’ practices, sarcoma clinical management involves many medical specialties and practitioners who are even less aware of the risks linked to sarcoma. It also appears to be more difficult to involve all of the actors implied in sarcoma clinical management, considering that they outnumber pathologists and most will only treat sarcoma a few times in their career. The difficulty of making healthcare professionals more sensitive to the specificity of these tumors is a common issue in all rare cancers.

Mapping the networks’ activity enabled us to observe the geographical coverage of these networks. Each regional expert center of these networks appeared as a hub providing expertise to most of the patients in its region, whereas national referral centers had a larger, almost national coverage. This illustrates the ability of the referral sarcoma networks to provide expertise to all sarcoma patients, even the most remote ones, and reduce geographical inequalities. Nevertheless, we will need a more qualitative assessment to take into account the delay in access to expertise.

With regard to the literature on the impact of travel distance and deprivation on the probability of being referred to expert centers, we hypothesize that some remote and deprived patients would have later access to expertise. Multivariate analysis like logistic regression will be conducted by ourselves to identify factors associated with optimal or later access to clinical and pathological expertise. These characteristic factors will be related to the patient (age, sex, medical history), cancer (histological subtype, grade, stage, size, depth), management (first examination contributing to diagnosis), or place of residence (distance to expertise, deprivation index, geographical classification). The socio-economic deprivation of district will be evaluated thanks to the European Deprivation Index, a French deprivation index used in cancer inequalities research [21]. Regarding the remoteness analysis, the data from the networks did not allow us to study the disparities inside urban districts. However, this precision loss is bound to the nationwide dimension of this project, which allows an original and quite exhaustive analysis of the access to sarcoma expertise in France that is based on a database containing tens of thousands of patients crossing clinical and geographical data. We will also produce a geographical classification to analyze sarcoma outcomes according the patients’ geographical context. Geographical classification (K-means classification) will be performed using 17 variables estimating environment quality, socio-demographic characteristics, and health care access in each French commune (*n* = 35,798 communes). In addition, some clinical variables such as the percentage of R2 surgery, inclusion of a patient in a clinical trial, or access to molecular biology examination will be tested to evaluate the clinical impact on patients of this unequal access to expertise.

This delayed access may lead to survival inequalities as patients may have not received the right treatment in the initial phase. After care follow-up will be performed for IGéAS cohort patients diagnosed in 2013. Diagnoses of intermediate malignity tumors, GIST, and low grade sarcoma are excluded from survival studies in order to focus on worse prognosis patients. The population of this nationwide survival study is estimated to be around 2200 patients. Moreover, upcoming data analysis will be useful for better knowledge of the functioning of national sarcoma networks by pointing out the least covered populations and the main impediments to accessing expertise. A survey with outside practitioners who are likely to refer patients to these networks will be conducted to improve our knowledge regarding impediments to accessing national sarcoma networks. In contrast to other countries, early access to referral network centers is recommended by practitioners in France, but no sanctions are planned if they do not comply. Considering the liberty of practitioners in France to refer patients to referral centers, improving early access to expertise and reducing inequalities requires measuring how these outside practitioners perceive and experience these networks. This survey will take place in the Auvergne–Rhône–Alpes and Grand Est regions thanks to the collaboration of regional cancer networks (French Réseaux Régionaux de Cancérologie), which know very well who the care actors are in their respective fields. Seven meetings, gathering together different care actors implied in sarcoma management (surgeons, pathologists, oncologists, radiologists, etc.) who work in public or private facilities, are currently being planned in different cities. This assessment of the networks by outside practitioners will provide some information about their needs and concerns regarding the current situation. This will also help steering committees design efficient recommendations to improve the experience of the networks by practitioners and their optimal use of this service.

Our field survey will also improve the assessment of these networks by bringing up the perception and experiences of this service by outside practitioners. Results of the data analysis and qualitative survey will be shared with all of the project participants and discussed in two steering committees. These committees will aim to draft a nationwide action plan to reduce inequalities in the access to expertise. Collaboration with referring sarcoma practitioners of these national networks and experiences from regional general cancer networks involved in the patient pathway improvement is a great asset of this project which will drive several actors to talk about the best way to improve access to expertise. The various analyses of data will help to identify the least covered populations and the clinical consequences of this low coverage. This information should establish appropriate responses that are essential to the various project participants who are also involved in the organization of sarcoma management. The action plan will be implemented by the national sarcoma networks by sharing guidelines nationwide.

## 5. Conclusions

The first results of the IGéAS research program showed the ability of the French sarcoma networks to provide access to expertise even to the most remote patients. As the literature reports that access to expert centers affects cancer survival, referral networks may have the potential to reduce cancer inequalities. Outside practitioners who have to refer their patients to expert centers have a major role in the success of this service. An upcoming survey will measure their perception of the referral networks and help us to improve their experience of the networks.

As further analysis will measure early access and survival inequalities, this research program may deliver some lessons about this service which will provide access to expertise without forcing patients to travel to expert centers and do not decree where patients have to be treated. It may also contribute to a broader reflection about the organization of cancer care where France and other European countries are struggling to reduce cancer inequalities.

## Figures and Tables

**Table 1:** Description of the IGéAS cohort.

**IGéAS Cohort**	**20,590**	
**Sex**		
Male	10,475	(50.9%)
Female	10,115	(49.1%)
**Age at Diagnosis**		
Mean (Std)	57.2 (20.5)	
Median (min; max)	60.5 (0.0; 105.2)	
**Geographic Origin**		
Mainland France	20,102	(97.6%)
Overseas territories	488	(2.4%)
**Type of Tumor**		
Soft tissue	13,081	(63.5%)
Viscera	5170	(25.1%)
Bone	2339	(11.4%)
**Histotype**		
***Sarcoma***	12,562	(61.0%)
Leiomyosarcoma	2249	(10.9%)
Undifferentiated sarcoma	2116	(10.3%)
Liposarcoma	1986	(9.6%)
Miscellaneous sarcomas	1794	(8.7%)
Other sarcomas	801	(3.9%)
Chondrosarcoma	715	(3.5%)
Osteosarcoma	620	(3.0%)
Myxofibrosarcoma	568	(2.8%)
Rhabdomyosarcoma	484	(2.4%)
Synovial sarcoma	386	(1.9%)
Malignant peripheral nerve sheath tumor	258	(1.3%)
Non classified sarcoma	585	(2.8%)
***Tumor of intermediate malignancy***	5343	(25.9%)
Intermediate fibroblastic/myofibroblastic tumors	2722	(13.2%)
Atypical lipomatous tumor	796	(3.9%)
Intermediate tumors of uncertain differentiation	635	(3.1%)
Intermediate vascular tumors	612	(3.0%)
Intermediate fibrohistiocytic tumors	334	(1.6%)
Other tumors of intermediate malignancy	124	(0.6%)
Intermediate chondrogenic tumors	66	(0.3%)
Intermediate osteogenic tumors	23	(0.1%)
Non classified tumor of intermediate malignancy	31	(0.1%)
***GIST***	2685	(13.0%)
**Year of Diagnosis**		
2011	4594	(22.3%)
2012	5041	(24.5%)
2013	5372	(26.1%)
2014	5583	(27.1%)
**Type of Reviewed Sampling**		
Tumor resection	9376	(45.5%)
Microbiopsy	4489	(21.8%)
No expert review	3777	(18.3%)
Open biopsy	2945	(14.3%)
Curettage	3	(0.0%)
**Days between Date of Sampling and Date of Review**		
Mean (Std)	43.4 (125.9)	
**Access to Pathological Expertise**		
Optimal access	11,841	(57.5%)
Late access	4972	(24.1%)
No access	3777	(18.3%)
**Clinical Situation at First MTB**		
No expert MTB discussion	7227	(35.1%)
Complementary treatment	4710	(22.9%)
Before surgery	3794	(18.4%)
After treatment	1300	(6.3%)
Before neo-adjuvant treatment	1283	(6.2%)
Before biopsy	1240	(6.0%)
After progression	1031	(5.0%)
Unknown	5	(0.0%)
**Access to Clinical Expertise**		
Optimal access	6318	(30.7%)
Late access	7045	(34.2%)
No access	7227	(35.1%)

**Table 2:** Remoteness and geographical inequalities in the access to sarcoma expertise for patients from mainland France.

Travel Time Distance to Expert Centers		Pathological Review	MTB Discussion
Number of Communes with expert centers		38	21
Median travel time (in minutes)		80.5	82.5
Average travel time (in minutes)		83.0	85.9
Average travel time (in minutes) by quintiles	*Q1*	41.2	43.1
	*Q2*	65.7	67.9
	*Q3*	81.4	83.7
	*Q4*	97.2	99.6
	*Q5*	131.6	137.2
